# Prognosis prediction of head and neck squamous cell carcinoma through the basement membrane-related lncRNA risk model

**DOI:** 10.3389/fmolb.2024.1421335

**Published:** 2024-10-23

**Authors:** Wenchao Bu, Mingguo Cao, Xinru Wu, Qiancheng Gao

**Affiliations:** School of Medicine, Lishui University, Lishui, Zhejiang, China

**Keywords:** head and neck squamous cell carcinoma, basement membrane-related genes, lncRNA, immune infiltration, data mining

## Abstract

**Background:**

Head and neck squamous cell carcinoma (HNSCC) ranks among the most widespread and significantly heterogeneous malignant tumors globally. Increasing evidence suggests that the basement membrane (BM) and associated long non-coding RNAs (lncRNA) are correlated with the onset of HNSCC and its prognosis. Our study aims to construct a basement membrane-associated lncRNAs (BMlncRNAs) marker to accurately predict the prognosis of HNSCC patients and find novel immunotherapy targets.

**Methods:**

The Cancer Genome Atlas (TCGA) database was accessed to acquire the transcriptome expression matrices, somatic mutation data, and clinical follow-up data of HNSCC patients. Utilizing co-expression analysis, the BMlncRNAs were identified and the differentially expressed lncRNAs (DEBMlncRNA) were then filtered, The filtering thresholds are FDR<0.05 and |log2FC|≥1. Furthermore, univariate analysis, least absolute shrinkage and selection operator (LASSO), and multivariable Cox regression were utilized to develop the risk model. The model then underwent thorough evaluation across diverse perspectives, encompassing tumor immune infiltration, tumor mutation burden (TMB), functional enrichment, and chemotherapy sensitivity.

**Results:**

The risk assessment model consists of 14 BMlncRNA pairs. The acquired data is indicative of the reliability of the risk score in its capacity as a prognostic factor. Individuals at high risk exhibited a poorer prognosis, and a statistically significant variance was noted in TMB and tumor immune infiltration compared to the low-risk group. Additionally, heightened sensitivity to paclitaxel and docetaxel was evident in the patients at high risk.

**Conclusion:**

We have established a BMLncRNA-based prognostic model that can provide clinical guidance for future laboratory and clinical studies of HNSCC.

## 1 Introduction

The characteristics of head and neck squamous cell carcinoma (HNSCC) include the presence of a multi-source malignant tumor group. HNSCC primarily originates from the squamous epithelial cells of the pharynx, oral cavity, and larynx. Globally, head and neck tumors are the sixth most common malignant tumors, with HNSCC being the most prevalent, accounting for 90% of head and neck tumors (Bray et al., 2018; Mody et al., 2021). The major causes of HNSCC in most patients are high-risk human papillomavirus (HPV) infection, genetic factors, excessive consumption of alcohol and tobacco use (Marur and Forastiere, 2016). Treatment approaches to HNSCC over the last few decades have typically included surgery, chemotherapy, radiotherapy, targeted therapy, emerging immunotherapy, and combination therapies. Although recent advancements have resulted in substantial improvement in the outcomes of individuals with HNSCC, the 5-year survival rate stands at a relatively low level of 50% (A et al., 2018; Canning et al., 2019). Therefore, it is imperative to delve into the processes governing the initiation and progression of HNSCC in depth. Equally as crucial is developing novel prognostic risk models that are both effective and reliable in the management of such cancers, discovering new biomarkers facilitating the prognosis prediction of HNSCC patients, and identifying promising therapeutic targets for HNSCC.

The basement membrane (BM) is predominantly present as a thin membrane formed between the basal surface of epithelial cells and the connective tissue. The BM is primarily composed of laminin and type IV collagen, along with various other molecules such as proteoglycans, growth factors, and others. The BM is involved in cell signaling, guiding cell migration and adhesion, maintaining structural integrity, and serving as a barrier for cells and large molecules. In epithelial cancer and carcinoma, cancer cell metastasis and invasion must pass through the structure of the BM, which physically prevents the invasion of cancer cells into the surrounding connective tissues. During the process of metastasis, the BM at the basal surface of lymphatic endothelial cells and vascular endothelial cells prevents the invasion and outflow of cancer cells. About 90% of tumor-related deaths are associated with this phenomenon (Fidler et al., 2017). The invasion of malignant cells into the BM is influenced by three major factors: matrix stiffening, contraction forces of epithelial cell scaffolding, and growth factor/cytokine signaling. Both matrix stiffening and epithelial contraction forces contribute to growth factor and cytokine signaling pathways, which are associated with promoting BM invasion (Chang et al., 2017). Additionally, the BM is involved in tumor angiogenesis. Key genes that encode BM proteins and cell surface interactors (CSI) are recognized as closely related to cancer progression (Jayadev et al., 2022). Any disruption in the expression of the components critical for the structural integrity of the BM has been linked with various diseases, particularly the invasion and metastasis of tumors (Wiradjaja et al., 2010). Specifically, in gastric cancer, dysregulation of the BM promotes tumor migration and invasion (Peng et al., 2010). Investigating the structure and processes of BM, as well as understanding how cancer cells invade it, holds the potential to drive the development of advanced technologies aimed at inhibiting cancer growth and metastasis.

Long non-coding RNAs (lncRNA) contain approximately 200 to 100,000 nucleotides and lack protein-coding capabilities. LncRNAs, along with circRNAs, can competitively bind to miRNA, inhibiting the binding of miRNA to mRNA, thereby regulating mRNA expression. Additionally, lncRNA can regulate chromatin function, modulate nucleosomes, and alter mRNA stability and translation, ultimately affecting gene expression in various signaling pathways (Hu and Messadi, 2023). Through the regulation of gene expression, lncRNAs are pivotal in biological processes. The abnormal expression of lncRNAs is implicated in the initiation, progression, and metastasis of tumors (Kong et al., 2018). Research has shown that lncRNAs are dysregulated in different types of tumors, promoting or inhibiting tumor progression by regulating gene expression. For instance, lncRNA BCAR4 targets the wnt signaling pathway via miR-370–3p to promote bladder cancer proliferation, survival, and metastasis (Zhang et al., 2020). Research indicates that overexpression of CYP4A22-AS1 in human LUAD cell lines promotes the proliferation and migration of LUAD cells. These overexpressed CYP4A22-AS1 can then downregulate miR-205–5p and miR-34c-5p, activating relevant signaling pathways, ultimately leading to LUAD proliferation and metastasis (Dong et al., 2023). LncRNAs hold promise as potential therapeutic targets and biomarkers for cancer (Chen et al., 2022). Additionally, aberrant lncRNA expression can function as a prognostic indicator for various cancers (Sun et al., 2022; Xiang et al., 2023; Xu et al., 2023).

However, limited research exists regarding the relationship between BM-related lncRNAs (BMlncRNA) and targets in HNSCC. To our knowledge, the prognostic relevance of BMlncRNAs and their association with the immune landscape of HNSCC remains incompletely understood. We aim to fill this gap by establishing a novel set of BMlncRNA features, which would aid in assessing the prognosis of afflicted individuals and understanding the immune landscape of HNSCC.

In order to develop a robust prognostic model and determine the relevance of BM-related genes in HNSCC, lncRNAs exhibiting differential expressions in the BM were identified. Subsequently, 14 types of BMlncRNAs were then utilized in constructing the risk feature. The established model holds promise in improving the reliability of prognostic risk stratification and aids in the formulation of treatment measures. This research utilized functional enrichment analysis to delve into the mechanisms through which BMlncRNAs influence the occurrence of HNSCC and its progression. Furthermore, the association of the risk score with various parameters was assessed for a more comprehensive analysis. These included the clinical pathological features, chemotherapy sensitivity, tumor mutation burden (TMB), and infiltration levels of immune cells. By delving deeper into the predictive relevance of lncRNAs, this research not only aids in the identification of novel therapeutic targets but also contributes to the development of effective drugs for individuals with HNSCC.

## 2 Materials and methods

### 2.1 Data acquisition

Transcriptomic profiles and clinical features of individuals with HNSCC were accessed at The Cancer Genome Atlas (TCGA, https://portal.gdc.cancer.gov/) database. In this study, 563 samples, comprising 44 non-tumor and 519 HNSCC tumor samples were analyzed. Clinical pathological data retrieved for individuals with HNSCC encompassed gender, age, stage, grade, TMN classification, survival status, and survival time. Strawberry Perl (v 5.30.0.1–64bit, https://strawberryperl.com/) was employed for the isolation of relevant data, including Fragments Per Kilobase Million (FPKM) and comprehensive pathological details, from every clinical sample. Subsequently, the programming language Perl was applied to differentiate lncRNA and mRNA within the HNSCC expression matrix. Additionally, single nucleotide variant (SNV) data and somatic mutation data were accessed at the TCGA database for computing the mutation burden of HNSCC. Ultimately, a set of 224 genes related to the BM was compiled from pertinent literature, referencing specific sources (Jayadev et al., 2022; Li et al., 2023) for the identification of BM-related genes.

### 2.2 Identification of BMlncRNA

Based on differentially expressed BM-related genes, relevant lncRNAs were identified from lncRNA expression data using Pearson’s correlation analysis (|R2|>0.4, *p* < 0.001) (Kuemmerlen et al., 2020). Subsequently, the interaction network between lncRNA and genes was visualized using R software. DEBMlncRNAs were obtained via the R “limma”, with FDR<0.05 and |log2FC|≥1 (Robinson et al., 2010) acting as the screening conditions.

### 2.3 Development of prognostic features of BMlncRNAs

To obtain BMlncRNAs linked with survival, the lncRNAs acquired in the preceding step underwent univariate Cox regression analysis. To mitigate the overfitting of prognostic features, our selection was further refined through the Least Absolute Shrinkage and Selection Operator (LASSO) regression analysis. Ultimately, a prognosis model associated with ERS was developed through the multivariable Cox analysis. Multiple R packages, encompassing “survival,” “caret,” “glmnet,” “survminer,” and “timeROC” were utilized for executing these analyses and visualizing the results. The risk score was computed as mentioned: risk score = ∑ [Exp (lncRNA) × coef (lncRNA)], where lncRNA coef signifies the coefficient of survival-related lncRNA, exp (lncRNA) indicates the level of expression of lncRNA, and coef (lncRNA) signifies the regression coefficient. Utilizing the median risk score, the cases under study underwent stratification into high-risk and low-risk groups for further investigation.

### 2.4 Association of the risk score with clinical features

Kruskal–Wallis and Wilcoxon assessments were employed to examine the relationship of risk scores with clinical features, like age, gender, AJCC staging, as well as T, N, and M staging.

### 2.5 Survival analysis of risk score

In order to examine the prognostic relevance of BMlncRNA prognosis, Kaplan-Meier (KM) methods and log-rank tests were utilized to examine the variance in survival across the risk groups.

### 2.6 Development and evaluation of nomogram

The R “rms” was utilized to develop a nomogram, which incorporated clinical features employed for predicting the survival of individuals with HNSCC. Calibration curves were employed to determine the prognostic prediction accuracy of the nomogram and calculate the Concordance Index (C-index) for the nomogram model. The R “timeROC” (Blanche et al., 2013) was employed to study the nomogram model, analyzing the Receiver Operating Characteristic (ROC) curve and the Area Under the Curve (AUC) values. Statistical analysis in this research was carried out via the R programming language. In order to aid in clinical decision-making, the risk scores and other clinical and pathological factors were combined to build the nomogram, which serves as a quantitative tool for assessing clinical outcomes. Calibration curves were used to examine the prognostic performance.

### 2.7 Principal component analysis and pathway enrichment analysis

The visualization of the expression patterns of lncRNAs related to BM in HNSCC samples was executed through the Principal Component Analysis (PCA) via “scatterplot3d”. When selecting differentially expressed genes (DEGs) between the two risk groups, specific criteria were considered (criteria: FDR q < 0.05; |log2FC| > 1). Results were acquired using “limma”. This was followed by enrichment analyses through Gene Ontology (GO) and Kyoto Encyclopedia of Genes and Genomes (KEGG). The assessment was executed through the packages “clusterProfiler” (Wu et al., 2021) and “ggplot2”. The DEGs that were identified were utilized to assess the risk model and investigate the functional pathways linked with HNSCC.

### 2.8 Evaluation of the association between Tumor-Infiltrating Immune Cells and risk score

The CIBERSORT (Newman et al., 2015) was utilized for assessing the Tumor-Infiltrating Immune Cells (TIICs) in HNSCC individuals in the TCGA cohort and computing their abundance. This was done with the aim of investigating the association of the risk scores with TIIC features. Furthermore, the Wilcoxon test was employed to examine the variation in immune cell abundance across the high-risk and low-risk groups, with results presented using box plots. The risk groups were comparatively assessed by computing the tumor microenvironment (TME) score, encompassing immune, stromal, and estimate scores.

### 2.9 Analysis of potential drug sensitivity and tumor mutation burden assessment

Tumor Mutation Burden (TMB) data was retrieved from TCGA (https://portal.gdc.cancer.gov/, accessed on 25 September 2023). Additionally, TMB differential analysis and TMB survival analysis were utilized to understand whether TMB affects the accuracy of the prognosis model. The Tumor Immune Dysfunction and Exclusion (TIDE) method was employed for predicting the immune response. Furthermore, in order to assess the effectiveness of the potential drugs, R (oncoPredict) was utilized to determine their IC50 values in the HNSCC risk groups (Maeser et al., 2021).

### 2.10 Statistical analysis

R software for data analysis and visualization (version 4.20). Co-expression analysis was performed using the “limma” package to obtain BMlncRNAs. The Wilcoxon test was used to compare the data between the 2 groups. Identification of lncRNAs in prognostic models using univariate and multivariate Cox analysis. The chi-square test is used to compare categorical data between groups. Correlation analysis was performed using the Pearson method. A *p*-value of <0.05 was considered statistically significant (^*^ *p* < 0.05, ^**^ *p* < 0.01, and ^***^ *p* < 0.001, ns, No significance.).

## 3 Results

### 3.1 Identification of BM-related lncRNA

**FIGURE 1:**
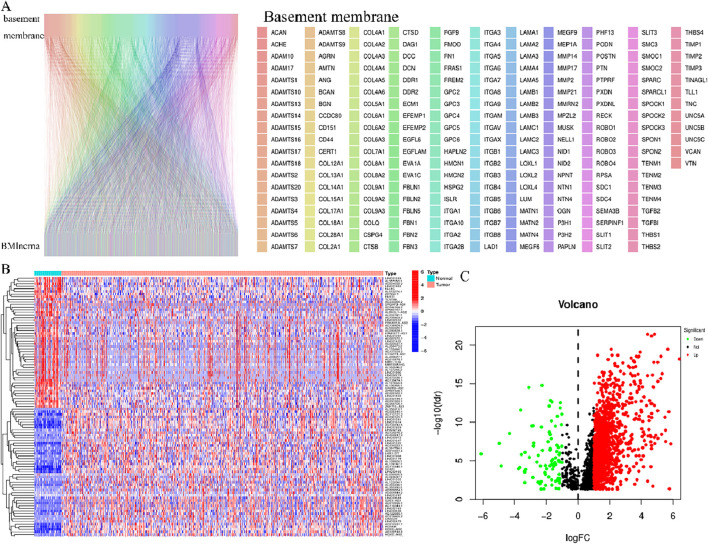
BM and basement membrane-associated BMlncRNAs in HNSCC **(A)** Sankey of BM and BM basement membrane genes and basement membrane-associated lncRNAs. Rectangles of different colors represent different BMs. **(B)** Heatmap showing differential expression of BMLncRNAs. Red represents high expression, and blue indicates low expression. **(C)** Volcano map showing BMLncRNA. The red dots represent the up regulated DEBMLncRNAs, the green dots represent the down-regulated DEBMLncRNAs, and the black dots represent the BMLncRNAs that are not significantly differentially expressed.

The Pearson test aimed to establish the correlation among BMlncRNAs. A total of 1983 lncRNAs associated with the BM were identified in HNSCC (Figure 1A), Their ligation to BMLncRNAs was visualized using a Sankey plot (Figure 1A), where different colors represent different BMs and curves linked BMLncRNAs. Utilizing a |correlation coefficient| > 0.4 and *p* < 0.001. Subsequently, 1,340 DEBMlncRNAs were identified, and the ones ranked in the top 20 upregulated and downregulated lncRNAs were displayed in relation to BM genes (Figure 1B). The volcano plot displayed differential lncRNAs associated with BM genes (Figure 1C).

### 3.2 Development of risk model

**FIGURE 2:**
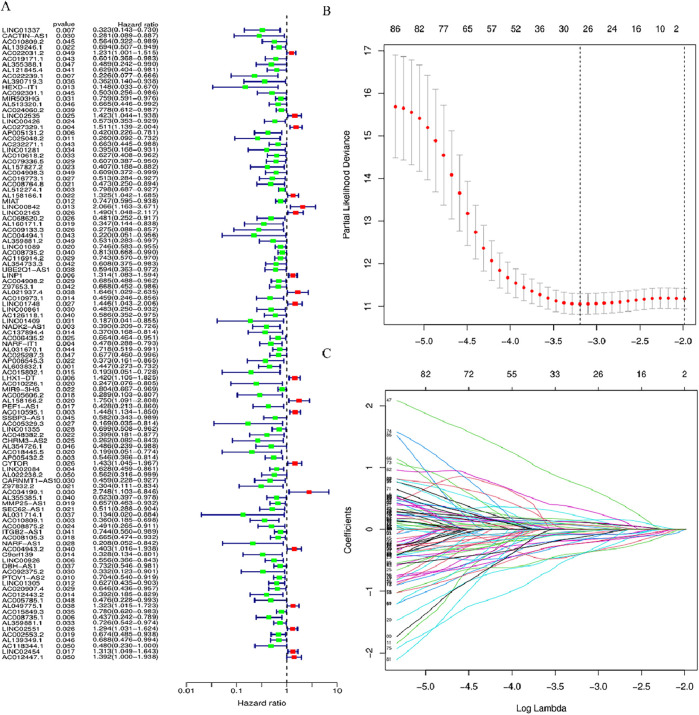
LncRNAs associated with BM prognosis. **(A)** Forest plot showing 102 lncRNAs linked with cuproptosis, where red signifies high-risk lncRNAs and green signifies low-risk lncRNAs **(B, C)** Lasso-Cox regression analysis was performed on DGBLncRNA to construct a prognostic prediction model. Each plot in Figure 2C represents the trajectory of change for each coefficient of the independent variable (in this study, representing the LncRNA). The ordinate is the value of the coefficient, the lower abscissa is the logarisa (λ), and the upper abscissa represents the number of non-zero coefficients in the model at this point.

The identification of the lncRNAs linked with prognosis was facilitated by integrating the survival data of individuals with HNSCC with the expression data of 1983 lncRNAs. Among the 519 HNSCC samples, 259 underwent random assignment to a test set and 260 to a training set. There was no statistically significant variance observed in terms of clinical features across the two groups of patients (*p* > 0.05) (Table 1). Univariate Cox analysis revealed 102 prognosis-related lncRNAs (Figure 2A). For further refining the gene set employed for developing the model, LASSO analysis was carried out (Figures 2B,C). Subsequently, multivariable Cox analysis was conducted, leading to the selection of 14 lncRNAs, ultimately utilized in constructing the risk model. The risk score for the patients was computed utilizing the formula mentioned earlier.

**TABLE 1:** Clinical information for all HNSCC patients.

Characteristics	Type	Total	Test	Train	*p*-value
Age	≤65	341 (65.7%)	173 (66.8%)	168 (64.62%)	0.6667
	>65	178 (34.3%)	86 (33.2%)	92 (35.38%)	
Gender	FEMALE	136 (26.2%)	70 (27.03%)	66 (25.38%)	0.7447
	MALE	383 (73.8%)	189 (72.97%)	194 (74.62%)	
Grade	G1	62 (11.95%)	28 (10.81%)	34 (13.08%)	0.0936
	G2	303 (58.38%)	140 (54.05%)	163 (62.69%)	
	G3	125 (24.08%)	72 (27.8%)	53 (20.38%)	
	G4	7 (1.35%)	5 (1.93%)	2 (0.77%)	
	unknow	22 (4.24%)	14 (5.41%)	8 (3.08%)	
Stage	Stage I	27 (5.2%)	15 (5.79%)	12 (4.62%)	0.7982
	Stage II	70 (13.49%)	32 (12.36%)	38 (14.62%)	
	Stage III	81 (15.61%)	37 (14.29%)	44 (16.92%)	
	Stage IV	266 (51.25%)	130 (50.19%)	136 (52.31%)	
	unknow	75 (14.45%)	45 (17.37%)	30 (11.54%)	
T	T0	1 (0.19%)	1 (0.39%)	0 (0%)	0.6847
	T1	48 (9.25%)	25 (9.65%)	23 (8.85%)	
	T2	135 (26.01%)	70 (27.03%)	65 (25%)	
	T3	99 (19.08%)	45 (17.37%)	54 (20.77%)	
	T4	174 (33.53%)	83 (32.05%)	91 (35%)	
	unknow	62 (11.95%)	35 (13.51%)	27 (10.38%)	
M	M0	185 (35.65%)	94 (36.29%)	91 (35%)	0.9914
	M1	1 (0.19%)	0 (0%)	1 (0.38%)	
	unknow	333 (64.16%)	165 (63.71%)	168 (64.62%)	
N	N0	175 (33.72%)	80 (30.89%)	95 (36.54%)	0.5019
	N1	67 (12.91%)	38 (14.67%)	29 (11.15%)	
	N2	169 (32.56%)	83 (32.05%)	86 (33.08%)	
	N3	8 (1.54%)	4 (1.54%)	4 (1.54%)	
	unknow	100 (19.27%)	54 (20.85%)	46 (17.69%)	

The risk score was computed as:

$$\begin{array}{ll} \text{risk score} & =\text{AC}022031.2\times \left(0.253004391422484\right)+\text{AC}022239.1\\ & \;\times \left(-1.15783343530458\right)+\text{AL}512274.1\\ & \;\times \left(0.280993884220756\right)+\text{LINP}1\\ & \;\times \left(0.338767951743789\right)+\text{AL}021937.4\\ & \;\times \left(1.25253156167544\right)+\text{AC}010973.1\\ & \;\times \left(0.823007676653033\right)+\text{LINC}01748\\ & \;\times \left(0.658875649899949\right)+\text{NADK}2-\text{AS}1\\ & \;\times \left(-0.763734240714477\right)+\text{AL}158166.2\\ & \;\times \left(0.464795341619062\right)+\text{AC}018445.5\\ & \;\times \left(-1.63436971235253\right)+\text{AP}005432.2\\ & \;\times \left(-0.497209980285157\right)+\text{LINC}02084\\ & \;\times \left(-0.524796620471794\right)+\text{AC}034199.1\\ & \;\times \left(0.818261361586273\right)+\text{AL}355385.1\\ & \;\times \left(-0.679732127938887\right) \end{array}$$

### 3.3 Association of risk score with clinical attributes

**FIGURE 3:**
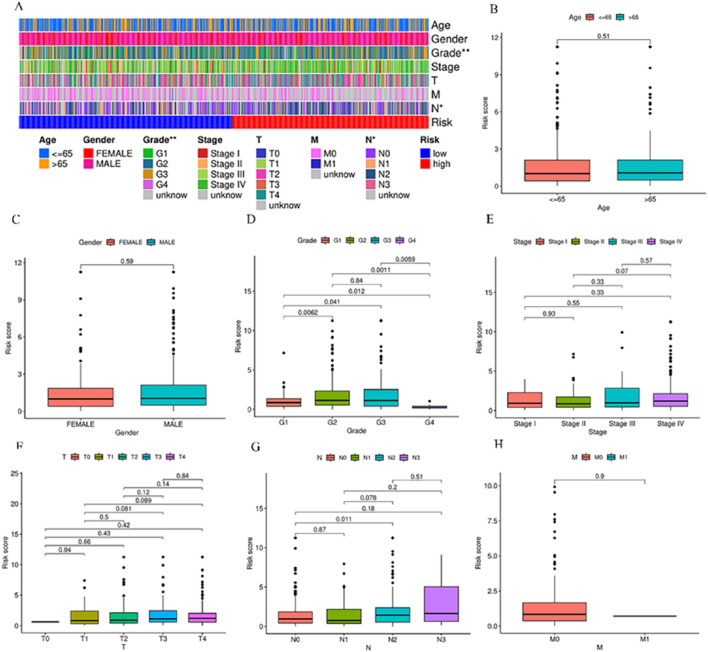
Relationship between various subgroups of the clinical features and risk scores. **(A)** Heat map of risk model and clinical features. **(B–H)** Differences in risk scores among patients in different subgroups under various clinical pathological features. ^*^ *p* < 0.05, ^**^ *p* < 0.01, and ^***^ *p* < 0.001, ns, No significance.

The link of risk score with clinical features was explored through limma and ggpubr. Bar plots and histograms reveal that the risk score is notably linked with clinical features (staging, N staging) (Figures 3A–H). With increasing risk scores, the distribution of various subtypes for every clinical feature in the samples (Figure 3A). The risk score of patients with G2 and G3 stages was higher than that of patients with G1 and G4 stages (*p* < 0.05) (Figure 3D), and the risk score of N2 stage was higher than that of patients with N0 (*p* < 0.05) (Figure 3G).

### 3.4 Survival analysis based on the risk model

**FIGURE 4:**
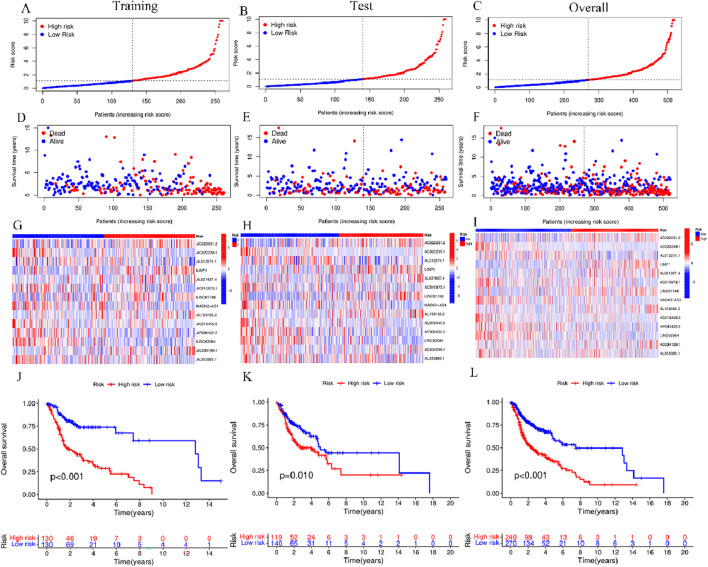
Risk model prognosis in different sets **(A–C)** Risk scores in the training, test, and complete sets for high- and low-risk groups. **(D–F)** Survival status in the three sets for the risk groups **(G–I)** Gene expression in the three sets for the risk groups. **(J–L)** Kaplan-Meier curves for assessing overall survival in the three sets for the risk groups.

HNSCC cases in TCGA were categorized into groups of high-risk and low-risk, with the intermediate risk score serving as the cutoff point for the training, test, and complete sets. The risk scores, gene expression, and survival status in these three sets are displayed in Figures 4A–I. As anticipated, significantly adverse overall survival (OS) was observed in high-risk HNSCC cases within the training (*p* < 0.001), the test (*p* = 0.01), and the complete sets (*p* < 0.001) (Figures 4J–L). The acquired data indicated an inverse relation between survival rate and risk, with high-risk HNSCC patients exhibiting a markedly lower rate of survival in contrast to their low-risk counterparts. This difference was evident in the distribution of risk score rankings and scatter plots, highlighting the correlation of patient survival status with risk scores, indicative of the heightened mortality rates with higher risk scores. The acquired data is indicative of the superior prognostic predictive capability of the newly developed risk model.

### 3.5 Validation of prognostic risk model accuracy for BMLncRNAs

**FIGURE 5:**
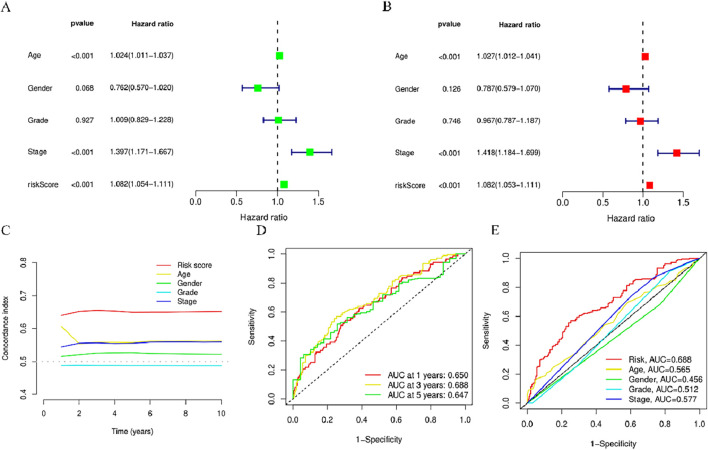
Assessment of the predictive risk model for BMlncRNAs and clinical attributes in the TCGA cohort. **(A)** Clinical attributes and risk scores in OS univariate analysis. **(B)** Clinical attributes and risk scores in OS univariate analysis. **(C)** Consistency index of risk scores and clinical attributes **(D)** ROC curves for assessing OS across 1-, 3-, and 5-years. **(E)** ROC curves for clinical attributes and risk scores.

**FIGURE 6:**
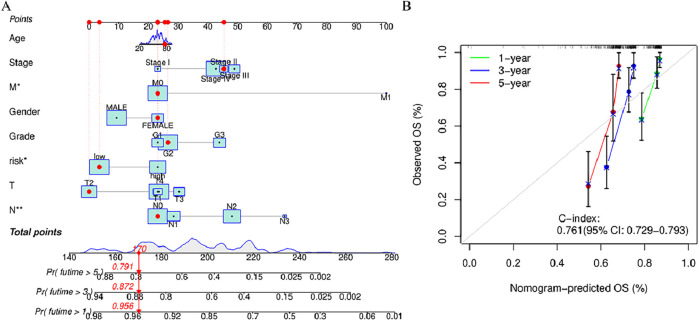
Nomogram and calibration curve of the model **(A)** Nomograms predicting 1-year, 3-year, and 5-year overall survival in patients with HNSCC **(B)** Calibration curves used to evaluate the accuracy of the nomogram model. The gray diagonal dashed line represents an ideal nomogram,The *x*-axis is the survival rate predicted by the nomogram, and the *y*-axis is the actual survival rate. ^*^ *p* < 0.05, ^**^ *p* < 0.01, and ^***^ *p* < 0.001, ns, No significance.

To explore the independent nature of the risk score as a survival risk factor for individuals afflicted with HNSCC, Cox models (univariate and multivariate) were established to examine the relation between these two elements. The univariate Cox findings showed that Age (*p* < 0.001, HR = 1.024)、Stage (*p* < 0.001, HR = 1.397) and riskScore (*p* < 0.001, HR = 1.082) were correlated with the HNSCC prognosis (Figure 6A). Moreover, Age (*p* < 0.001, HR = 1.027)、Stage (*p* < 0.001, HR = 1.418) and riskScore (*p* < 0.001, HR = 1.082) were identified as independent variables in the multivariate Cox analysis that affected HNSCCprognosis (Figure 6B). Over time, the consistency index of the risk score consistently exceeded that of any other clinical component, suggesting that the risk level holds greater accuracy concerning the outcome prediction of HNSCC (Figure 5C). Furthermore, ROC curve evaluation of the predictive performance of the risk score yielded respective AUCs of 0.650, 0.688, and 0.647 across 1 year, 3 years, and 5 years (Figure 5D), with a maximum AUC value of 0.688. The acquired data is indicative of its good sensitivity and specificity. These results indicate promising predictive capabilities of the risk model for the OS of individuals with HNSCC. The risk model yielded an AUC value of 0.688, surpassing age (0.565), gender (0.456), staging (0.512), and grading (0.577) (Figure 5E), demonstrating that the risk score outperforms other clinical pathological variables.

### 3.6 Joint risk score and clinical feature prediction of the survival rate and prognosis accuracy of HNSCC patients in the entire cohort using a column chart

When constructing the column chart to facilitate the prediction of the survival rate of individuals with HNSCC, both risk scores and clinical parameters (like gender, age, N stage, T stage) were considered, This nomogram provides an accurate prediction of OS in patients with HNSCC at 1, 3, and 5 years (Figure 6A). Calibration plot analysis shows that the curve predicted by the analysis closely follows the 45-degree line (ideal curve), C index 0.692, and 95% confidential interval (CI): 0.630–0.755,The calibration curves show good agreement between the nomogram and the predictions for 1-, 3-, and 5-year OS (Figure 5B). This indicates that the nomogram agrees well with the ideal model.

### 3.7 Prognostic analysis of risk score and clinical attributes

**FIGURE 7:**
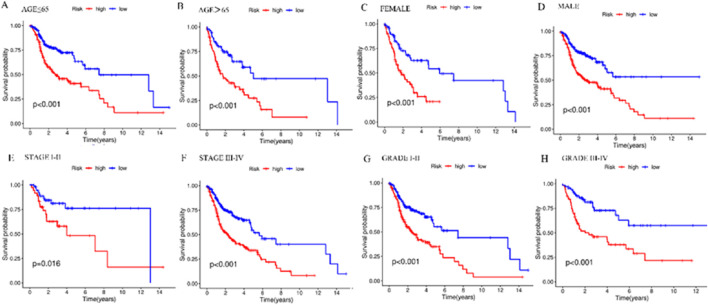
Kaplan-Meier survival curves in the high- and low-risk groups with different clinical features. **(A)** Kaplan-Meier survival curves in ≤65-year-old patients in different risk groups. **(B)** Kaplan-Meier survival curves in patients aged 65> years in different risk groups. **(C)** Kaplan-Meier survival curves in FEMALE patients in different risk groups. **(D)** Kaplan-Meier survival curves in MALE patients with different risk groups. **(E)** Kaplan-Meier survival curves in patients with STAGE I-II in different risk groups. **(F)** Kaplan-Meier survival curves in patients with STAGE III-IV in different risk groups. **(G)** Kaplan-Meier survival curves in patients with GRADE I-II in different risk groups. **(H)** Kaplan-Meier survival curves in patients with GRADE III-IV in different risk groups. ^*^ *p* < 0.05, ^**^ *p* < 0.01, and ^***^ *p* < 0.001, ns, No significance.

Stratified analysis was employed for the purpose of delving deeper into the prognostic relevance of risk scores within distinct clinical conditions. The outcomes indicate the presence of a statistically significant variance across the high-risk group and the low-risk group in terms of clinical feature subgroups (*p* < 0.05), with the lower-risk individuals exhibiting a favorable prognosis in contrast to their higher-risk counterparts (Figure 7). The results revealed that the risk model was applicable to patients with different clinical characteristics.

### 3.8 Functional enrichment of principal component analysis and BMlncRNA markers

**FIGURE 8:**
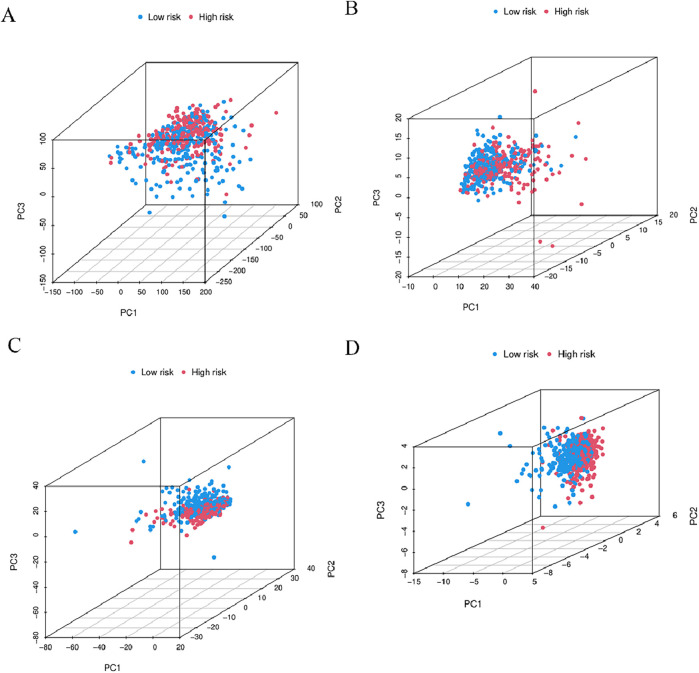
3D-PCA considered the different gene spectra of HNSCC patients. **(A)** Comparative gene modules, **(B)** gene modules related to BM, **(C)** BMlncRNA modules, **(D)** BMlncRNA prognostic modules can more clearly divide HNSCC cases into high-risk and low-risk groups. PCA = principal component analysis; The red and blue dots represent high-risk and low-risk gene.

**FIGURE 9:**
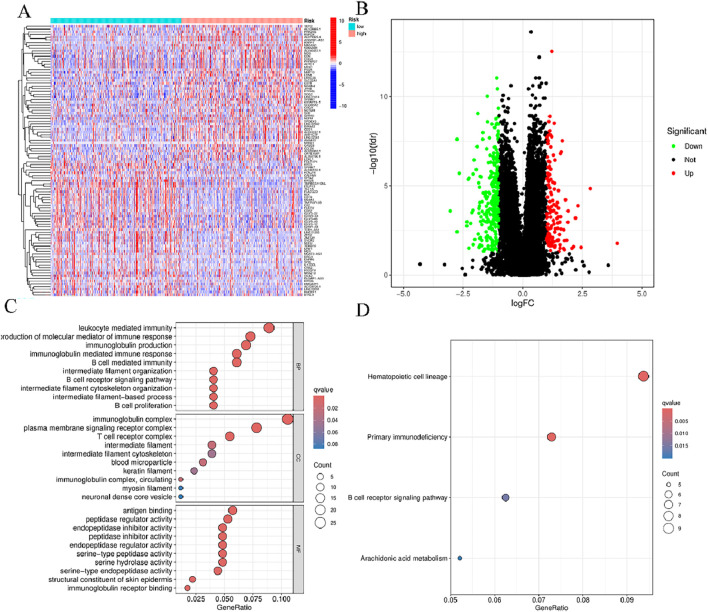
Functional enrichment of BMlncRNA features. **(A)** Heatmap: High-expression genes are depicted in red; low-expression genes are depicted in green. **(B)** Volcano plot: Each point represents a protein: downregulated (green), upregulated (red), and non-significant (black). **(C)** GO enrichment of genes with differential expression in cellular components (CC), biological processes (BP), and molecular functions (MF) **(D)** Enrichment analysis of differential genes between the two groups obtained after KEGG analysis.

In order to determine whether the prognosis features of BMlncRNA can divide HNSCC cases into high-risk and low-risk groups, PCA analysis was conducted. This investigation took into account the different gene spectra of HNSCC patients (Figure 8). The prognostic module utilizing the lncRNA related to the BM genes exhibits a greater functionality in clearly dividing HNSCC cases into high- and low-risk groups. To study the biological functions and pathways possibly associated with these risk groups, functional enrichment analysis was conducted on the DEGs. The acquired data indicated the existence of 405 DEGs across the two groups. The heatmap and volcano plots were utilized to visually represent the top 50 DEGs (Figures 9A, B). Additionally, the distribution of DEGs was determined in terms of functional enrichment levels using GO analysis method (focusing on biological processes [BP], cellular components [CC], and molecular functions [MF]) (Figure 9C). Multiple immune-linked biological processes exhibited enrichment, like immunoglobulin production, immunoglobulin-mediated immune response, and B cell-mediated immunity (*p* < 0.05). Results from KEGG analysis suggest significant enrichment of these DEGs in immune-related pathways (*p* < 0.05) (Figure 9D).

### 3.9 Correlation between risk scoring models and somatic variants

**FIGURE 10:**
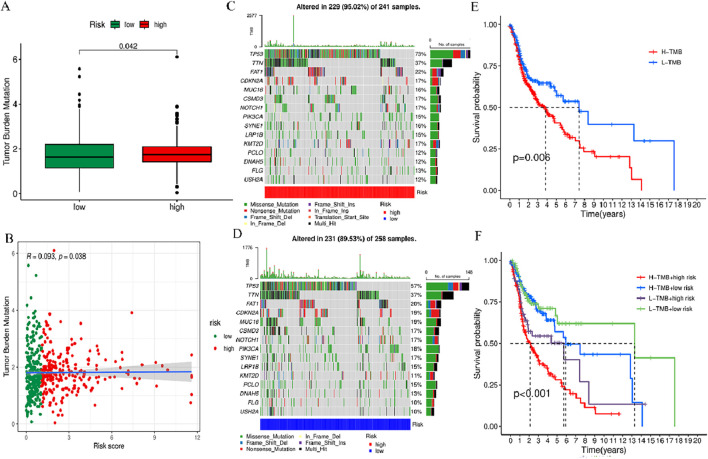
TMB analysis of prognosis features. **(A)** Box plot showing the TMB difference across high- and low-risk groups. **(B)** Correlation curve between TMB and risk score. **(C)** Waterfall plot of tumor mutation rates based on prognosis features in the high-risk group. **(D)** Waterfall plot of tumor mutation rates based on prognosis features in the high-risk group. **(E)** Kaplan-Meier (KM) curves of individuals with HNSCC across H-TMB and L-TMB groups. **(F)** KM curves of HNSCC patients in H-TMB + low-risk, H-TMB + high-risk, L-TMB + low-risk, and L-TMB + high-risk.

To comprehensively evaluate the 519 HNSCC samples in terms of the gene mutation spectrum, the R “maftools”, incorporating risk scores was employed for the analysis. The analysis revealed that the individuals at higher risk exhibited a heightened TMB in contrast to their lower-risk counterparts. The acquired data indicated the presence of a notably positive correlation of TMB with the risk score (Figures 10A, B). Waterfall plots were generated, taking into account the mutation frequency, for both risk groups. The acquired data implied that the higher-risk individuals displayed heightened mutation frequency in 15 genes in contrast to their lower-risk counterparts (Figures 10C, D). Among the mutated genes determined in the high-risk individuals, the first five were TP53 (73%), TTN (37%), FAT1 (22%), CDKN2A (17%), and MUC16 (16%). In comparison, the first five genes noted in the low-risk group were TP53 (57%), TTN (37%), FAT1 (20%), CDKN2A (19%), and MUC16 (19%). Furthermore, in comparison with the low-risk group, the expression of TP53 and FAT1 increased in the high-risk group. Furthermore, to investigate the potential of the risk score for predicting tumor burden survival, the recorded TMB values for individuals with HNSCC were examined. This was followed by the categorization of these individuals into high TMB (H-TMB) and low TMB (L-TMB) groups, per the median TMB. The analysis of the KM curves indicated that individuals in the L-TMB group were linked with a heightened likelihood of experiencing longer survival in contrast to the H-TMB group (log-rank, *p* = 0.006, Figure 10E). Furthermore, the survival analysis data of various subgroups indicate significant variance in survival outcomes among the H-TMB + low-risk, H-TMB + high-risk, L-TMB + low-risk, and L-TMB + high-risk groups (log-rank, *p* < 0.001, Figure 10F).

### 3.10 Estimation of tumor immune microenvironment, immune cell infiltration and immune-related functions, and drug sensitivity

**FIGURE 11:**
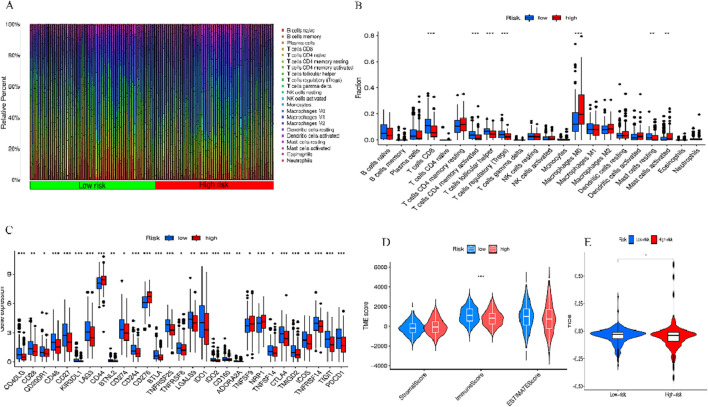
Two sets of immune features and cancer immunotherapy response. **(A)** Bar graph displaying the proportion of 22 immune cell types in terms of CC in patients of TCGA-HNSCC, with each column representing a sample ID. **(B)** Variation in the proportions of 22 immune cell types across the high- and low-risk individuals. **(C)** High- and low-risk immune checkpoints. **(D)** TME scores of the two groups. **(E)** Differential TIDE prediction between the two groups. ^*^ *p* < 0.05, ^**^ *p* < 0.01, and ^***^ *p* < 0.001, ns, No significance.

**FIGURE 12:**
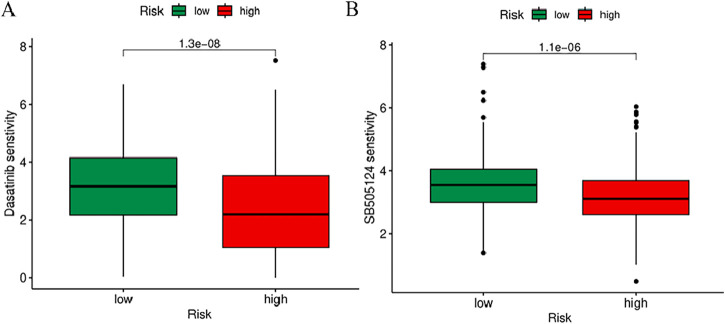
Prediction of sensitivity to chemotherapy drugs based on IC50 values. **(A)** Difference in IC50 in Dasatinib. **(B)** Difference in IC50 in SB505124.

To further explore the association between BM-related features in HNSCC patients and anti-tumor immunity, CIBERSORT was utilized to examine the immune cell invasion in individuals with HNSCC acquired from TCGA. The proportion of all typical immune cells is depicted in Figure 11A. Moreover, the proportions of various immune cells were comparatively assessed between the risk groups and statistically significant differences were found in macrophages M0, Mast cells activated, T cells CD8, T cells CD4 memory activated, T cells follicular helper, T cells regulatory (Tregs),and Mast cells resting across the two groups (*p* < 0.05) (Figure 11B). To discern variations in immune cell invasion across the risk groups, immune score (immune cell infiltration in tumor tissue), the stromal score (stromal cells in tumor tissue), and estimate score (sum of individual case stromal and immune scores) were comparatively assessed. Moreover, this analysis showed notably elevated stromal scores in the low-risk group (*p* < 0.001) (Figure 11C). In the low-risk group, there were more immune cells and better immune function scores. In the low-risk group, most immune checkpoints were more active (Figure 11D). Furthermore, immune therapeutic biomarkers were studied and it was concluded that the individuals at higher risk exhibited a stronger response to immunotherapy (*p* < 0.05) (Figure 11E). The IC50 values of two immune therapeutic drugs, including Dasatinib and SB505124, were noted to be diminished in the individuals at higher risk (*p* < 0.05) (Figure 12). The data acquired may contribute to clinical applications.

## 4 Discussion

Treatment measures for individuals with HNSCC involve chemotherapy, radiotherapy, targeted therapy, and a variety of other immunotherapies (Bishop et al., 2021). Despite significant advancement in HNSCC treatment, the prognosis for these individuals is relatively poor clinically. These adverse outcomes are attributable to the heightened rate of malignancy and metastasis, as well as increased heterogeneity observed in individuals with HNSCC. The 5-year OS rate in this study was observed to be lower and this diminished rate can be attributed to the absence of effective early diagnosis and resistance strategies (Johnson et al., 2020). Recognizing these challenges, constructing a predictive model specifically focused on the BM could provide vital information for effectively guiding the prognosis and treatment of individuals afflicted with HNSCC.

Therefore, tumors lacking BMlncRNA prognostic features. In this research, we attempted to establish a novel lncRNA feature to facilitate the prediction of the prognosis and immune microenvironment of HNSCC. To our knowledge, researchers have found BMlncRNAs across diverse cancers, and recently, attention has been drawn toward specifically exploring the relationship between BMlncRNAs and various cancers. There have been studies that have been successful in constructing different BM-related gene features to facilitate the prognosis prediction of bladder cancer, clear cell renal cell carcinoma, and hepatocellular carcinoma (Feng et al., 2022; Li et al., 2023; Jin et al., 2023).

LncRNAs represent promising biomarkers with therapeutic implications across diverse diseases, particularly in the context of tumors. Herein, 48 dysregulated cuproptosis-related lncRNAs were determined in the context of HNSCC. Additional screening aided in further refining the selection, resulting in the isolation of 14 BMLncRNAs (AC022031.2, AC022239.1, AL512274.1, LINP1, AL021937.4, AC010973.1, LINC01748, NADK2-AS1, AL158166.2, AC018445.5, AP005432.2, LINC02084, AC034199.1, AL355385.1). Qiu et al. (2022) constructed an 8-lncRNA prognostic model including AL512274.1, which is of significant value in prognosis prediction and immune assessment. LINP1 offers a crucial role in enhancing the proliferation and spread of pancreatic cancer cells by modulating miR-491–3p (Chen et al., 2020). AC010973.1 is highly correlated with poor prognosis in hepatocellular carcinoma. Furthermore, the observed upregulation of CDK5 expression by AC010973.1 has been identified as a factor contributing to the heightened proliferative, invasive, and migratory capacities of HCC cells (Li et al., 2020). LINC01748 heightens the invasiveness of NSCLC cells by competitively binding with miR-520a-5p, leading to HMGA1 overexpression (Tan et al., 2022). Sun et al. (2020) constructed five immune-related lncRNA features, among them LINC02084, demonstrating the potential to predict the prognosis of individuals with KIRC. Zhang et al. (2023) built an esophageal cancer prognostic model consisting of six lncRNAs, including AC034199.1, which has a better diagnostic value in comparison with other clinical features. Hou et al. (2022) developed a 15-lncRNA prognostic model including AP005432.2, which shows good efficacy in predicting the prognosis, immune response, and chemotherapy response in bladder cancer. The remaining seven BMlncRNAs (AC022031.2, AC022239.1, AL021937.4, NADK2-AS1, AL158166.2, AC018445.5, AL355385.1) have not been related to cancer based on existing reports. Therefore, additional investigation is necessary to unravel the prognostic mechanisms associated with these BMlncRNAs in HNSCC. In this study, we constructed BMlncRNAs signatures based on 14 BMlncRNAs through integrated bioinformatics analysis, which confirmed prognostic value and served as an independent prognostic factor and potential therapeutic target for patients with HNSCC.

The data required for the analyses were acquired via the TCGA database. Subsequently, the individuals under study were categorized into two risk groups per the median risk scores. PCA analysis demonstrated superior performance of BMlncRNA features in distinguishing the patients in contrast to whole-genome expression, BM-related genes, and BMlncRNAs. The KM curve demonstrated that individuals at higher risk exhibited shorter OS values relative to the individuals in the low-risk group. Additionally, Cox regression analysis (both univariate and multivariate) affirmed the independent predictive significance of risk scores for HNSCC patients. Moreover, a column chart was constructed by integrating the risk score and clinical pathological factors to examine the predictive accuracy and practical applicability. The risk model exhibited higher AUC and C-index values in contrast to those derived from clinical factors (gender, age, clinical stage, and grade). The data acquired suggests accurate prognosis prediction for individuals with HNSCC. It was observed that the developed features outperform traditional clinical markers in providing precise prognostic insights. Our studies thus provide a new scoring system for prognosis prediction in HNSCC patients.

Enrichment analyses, GO and KEGG, were utilized to explore the processes relevant to the risk model. The resulting data revealed a notable enrichment of DEGs, primarily associated with the immune response and pathways related to tumors. Further exploration of the correlation between the level of immune cell infiltration and the risk score was conducted. Using CIBERSORT, the immune cell infiltration status of all HNSCC patients acquired from the TCGA database was examined. Additionally, the study conducted a comparison of the proportions of various immune cells between the high-risk and low-risk groups, revealing statistically significant variance in macrophages M0,Mast cells activated, T cells CD8, T cells CD4 memory activated, T cells follicular helper, T cells regulatory (Tregs),and Mast cells resting. The research results indicate that the immune scores of low-risk individuals are markedly elevated than those of high-risk individuals. This phenotype suggests a potential inverse correlation between the risk score and the immune status.

Prior research indicates that TMB may function as a reliable biomarker (Topalian et al., 2016). TMB scores were computed as per TCGA somatic mutation data, and the high-risk group demonstrated elevated TMB scores. A strong association exists between the classifier index based on the BM and the TMB. Higher TMB scores are associated with poorer prognosis. Furthermore, both high and low TMB scores in the low-risk group are linked to better prognosis. Moreover, this research revealed the relevance of the lncRNA model associated with cuproptosis. The immune therapeutic biomarkers were investigated as well and it was concluded that the high-risk group exhibits a strong response to immunotherapy. Variations in IC50 values for various drugs among distinct HNSCC subgroups revealed two relevant drugs, Dasatinib and SB505124. Our study showed that the high-risk group had a higher sensitivity to dasatinib and SB505124 than the low-risk group. However, the accuracy and suitability of these personalized drugs need further research.

However, this study has some limitations. Enhanced reliability can be achieved through the utilization of additional validation sets to confirm prognostic values. Moreover, to acquire a more thorough insight into its mechanism of action, *in vitro* and *in vivo* experiments should be carried out. Therefore, we plan to conduct further research into these aspects and address these limitations.

## Data Availability

The datasets presented in this study can be found in online repositories. The names of the repository/repositories and accession number(s) can be found in the article/supplementary material.
